# Diagnostic performance of CMR, SPECT, and PET imaging for the detection of cardiac amyloidosis: a meta-analysis

**DOI:** 10.1186/s12872-021-02292-z

**Published:** 2021-10-07

**Authors:** Zhaoye Wu, Chunjing Yu

**Affiliations:** Department of Nuclear Medicine, Affiliated Hospital of Jiangnan University, Wuxi, China

**Keywords:** Cardiac amyloidosis, Noninvasive imaging, Radionuclide, Diagnostic performance, Meta-analysis

## Abstract

**Background:**

Noninvasive myocardial imaging modalities, such as cardiac magnetic resonance (CMR), single photon emission computed tomography (SPECT), and Positron emission tomography (PET), are well-established and extensively used to detect cardiac amyloid (CA). The purpose of this study is to directly compare CMR, SPECT, and PET scans in the diagnosis of CA, and to provide evidence for further scientific research and clinical decision-making.

**Methods:**

PubMed, Embase, and Cochrane Library were searched. Studies used CMR, SPECT and/or PET for the diagnosis of CA were included. Pooled sensitivity, specificity, positive and negative likelihood ratio (LR), diagnostic odds ratio (DOR), their respective 95% confidence intervals (CIs) and the area under the summary receiver operating characteristic (SROC) curve (AUC) were calculated. Quality assessment of included studies was conducted.

**Results:**

A total of 31 articles were identified for inclusion in this meta-analysis. The pooled sensitivities of CMR, SPECT and PET were 0.84, 0.98 and 0.78, respectively. Their respective overall specificities were 0.87, 0.92 and 0.95. Subgroup analysis demonstrated that ^99m^Tc-HMDP manifested the highest sensitivity (0.99). ^99m^Tc-PYP had the highest specificity (0.95). The AUC values of ^99m^Tc-DPD, ^99m^Tc-PYP, ^99m^Tc-HMDP were 0.89, 0.99, and 0.99, respectively. PET scan with ^11^C-PIB demonstrated a pooled sensitivity of 0.91 and specificity of 0.97 with an AUC value of 0.98.

**Conclusion:**

Our meta-analysis reveals that SEPCT scans present better diagnostic performance for the identification of CA as compared with other two modalities.

**Supplementary Information:**

The online version contains supplementary material available at 10.1186/s12872-021-02292-z.

## Background

Cardiac amyloidosis (CA) is a myocardial disease characterized by abnormal extracellular deposition of amyloid fibrils, which gives rise to a progressive structural and functional damage to the cardiac tissue [1, 2]. CA is the main cause of death and occurrence in systemic amyloidosis [3]. On the basis of the underlying nosology, two subtypes (systemic light chain (AL) amyloidosis and transthyretin (ATTR) amyloidosis) account for most cases of cardiac amyloid. The two types of amyloidosis possess different clinical presentations and prognosis [4, 5].

The diagnostic approaches of cardiac amyloidosis include clinical symptoms, laboratory tests, non-invasive imaging, and histopathological diagnosis [6]. Unfortunately, this disease is commonly asymptomatic over a period of time from the beginning and the symptoms are usually nonspecific, and therefore its diagnosis is often delayed [2]. Currently, the gold standard for the diagnosis of CA is endomyocardial biopsy [7]. Nevertheless, endomyocardial biopsy is an invasive modality which can lead to unwanted complications. Echocardiography is widely employed for the diagnosis of CA in patients with suspected amyloidosis in clinical settings, however, it does not differentiate ATTR from AL CA [8]. It is reported that the diagnostic accuracy of echocardiography in combination with electrocardiogram (ECG) findings is only 60% [9]. Cardiac magnetic resonance (CMR) imaging is a mature and advanced imaging approach to describe the morphological characteristics and function of the heart and determine the characteristics of cardiac tissue, however, it may be in lack of specificity in distinguishing the potential causes of different types of CA and holds important prognostic information [5, 10, 11]. Molecular imaging is another type of noninvasive modality for the diagnosis of CA. The favorable efficacy of technetium (Tc)-99m labelled bone seeking tracers in single photon emission computed tomography (SPECT) (pyrophosphate (^99m^Tc-PYP), 3, 3-diphosphono-1,2-propanodicarboxylic acid (^99m^Tc-DPD), and hydroxymethylene diphosphonate (^99m^Tc-HMDP)) for diagnosing CA have been manifested in several studies [12–14]. Furthermore, positron emission tomography (PET) scans with tracers including ^11^C-Pittsburgh compound B (PIB), ^18^F-florbetapir, ^18^F-florbetaben, ^18^F-NaF, and ^18^F-flutemetamol have been studied for cardiac amyloidosis [15–18]. Compared to SPECT, PET shows higher spatial resolution and may provide more accurate quantification of absolute tracer uptake [5, 14].

As far as we are concerned, accumulated studies and meta-analyses have evaluated diagnostic performance of non-invasive modalities for the confirmation of CA [12, 19–23]. Most of these meta-analyses are on single-modality basis. The aim of this study was to generate a more comprehensive comparison of CMR, SPECT, and PET in the identification of CA by pooling the data of available studies, and subsequently to provide updated evidence-based information and hints for not only scientific research but also for the implement and decision-making of clinical practitioners.

## Methods

This meta-analysis was conducted strictly on the basis of the Preferred Reporting Items for Systematic Reviews and Meta-analysis (PRISMA) [24]. Details on each procedure of the study were reported as follows.

### Search strategy and study selection

The researchers did a comprehensive search of the electronic databases: PubMed, Embase, and Cochrane Library from January 1, 2011 to November 30, 2020, only articles in the English language were considered. The following key words or phrases were used for the database research: “cardiac magnetic resonance”, “CMR”, “single-photon emission computed tomography”, “SPECT”, “positron emission tomography”, “PET”, “Cardiac amyloidosis” and “CA”. The references of these articles were also searched for potential eligible researches. The inclusion criteria of this meta-analysis were as follows: (a) CMR, SPECT and/or PET were employed for the detection of CA in patients with suspected or diagnosed CA; (b) specific gold standard reference was used to evaluate the diagnostic performance; (c) absolute numbers of patients with true positive (TP), false positive (FP), true negative (TN) and false negative (FN) outcomes were depicted directly in the original article or the references or all these numbers could be calculated based on the articles. In case that the studies were carried out by the same research team, only those with the largest sample size or the most complete information were included. Studied without necessary parameters mentioned above, case reports, reviews, letters to the editorial, conference abstracts, and animal studies were not taken into account in the meta-analysis.

Two authors independently conducted the database search and study selection. Discrepancies were resolved by discussion until a final decision was reached.

### Data extraction and quality assessments

Two reviewers independently performed the screening of types of articles, titles and abstracts according to the protocol of study selection, hereafter the full-text reading of the articles was conducted for the final inclusion. The following information was retrieved from each study included: name of first author, year of publication, number of patients analyzed, reference standard, type of detection modalities and type of radiopharmaceuticals used in the study, absolute number of participants with TP, TN, FP and FN results. Quality Assessment of Diagnostic Accuracy Studies-2 (QUADAS-2) criteria was used to assess the quality of each included studies, this quality scale includes components in terms of participant selection, index test, reference standard, as well as flow and timing [25]. Any disagreements occurred in the process of data extraction and quality assessments were resolved by consensus.

### Statistical analysis

Data were analyzed employing the Stata version 15.0 software and Review Manager version 5.3 software at the study level. A *p* value < 0.05 was considered to be statistically significant. We calculated pooled sensitivity, specificity, positive and negative likelihood ratio (LR), diagnostic odds ratio (DOR), and their respective 95% confidence intervals (CIs) and the area under the summary receiver operating characteristic (SROC) curve (AUC). The Cochran Q and the I^2^ statistics were introduced to assess the heterogeneity of studies included on qualitative and quantitative basis. I^2^ values within 0–25%, 25–50%, 50–75%, and 75–100% manifested insignificant, low, moderate, and high heterogeneity, respectively [26]. Funnel plots were conducted to qualitatively assess potential bias of publication, A Deeks’ method was used to statistically test the asymmetry of the funnel plots and detect publication bias [27]. Moreover, we used sensitivity analysis to evaluate the impacts of each single study on the pooled outcomes.

## Results

### Study selection and characteristics

**Fig. 1:**
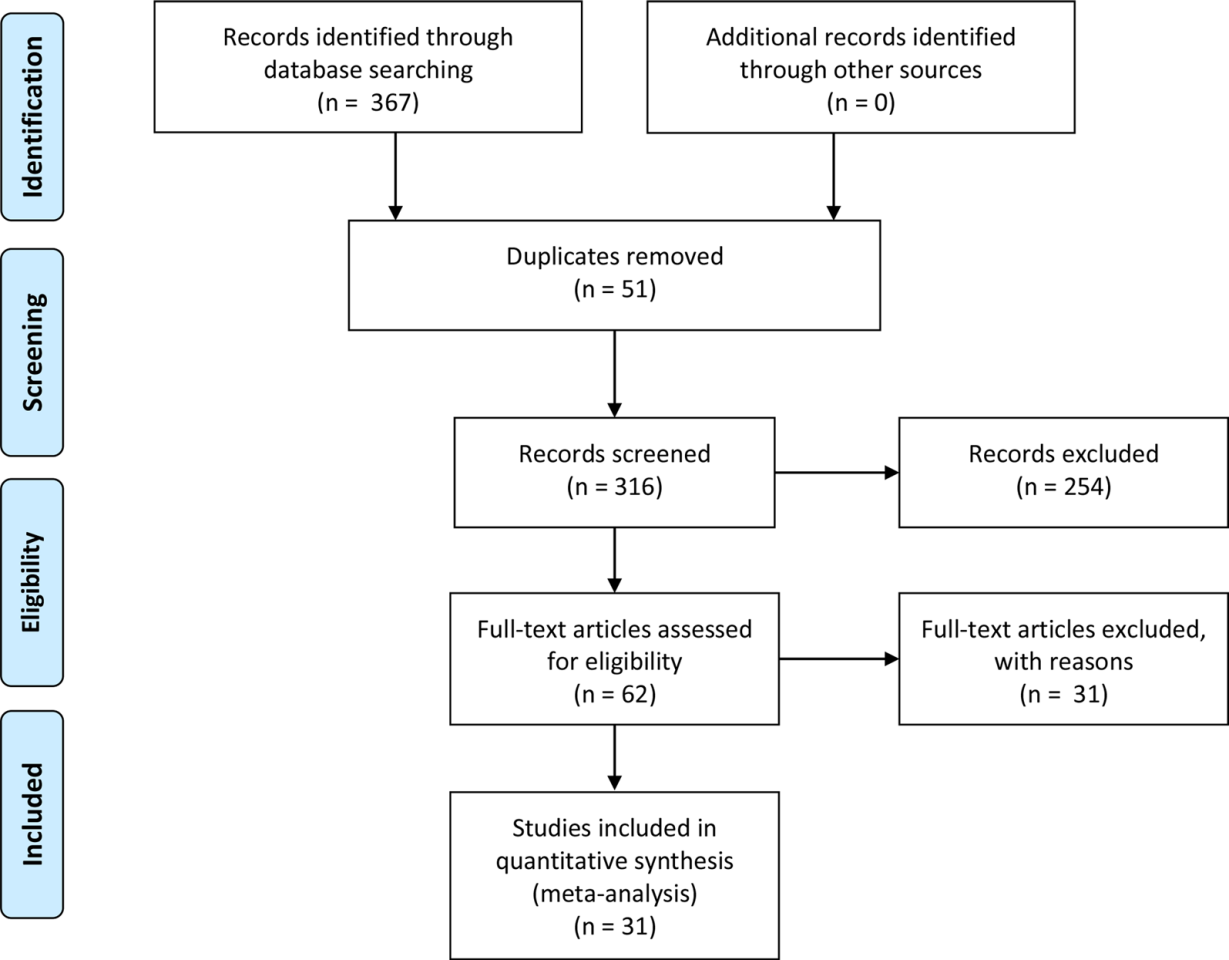
Search results and flow chart of the meta-analysis

**Fig. 2:**
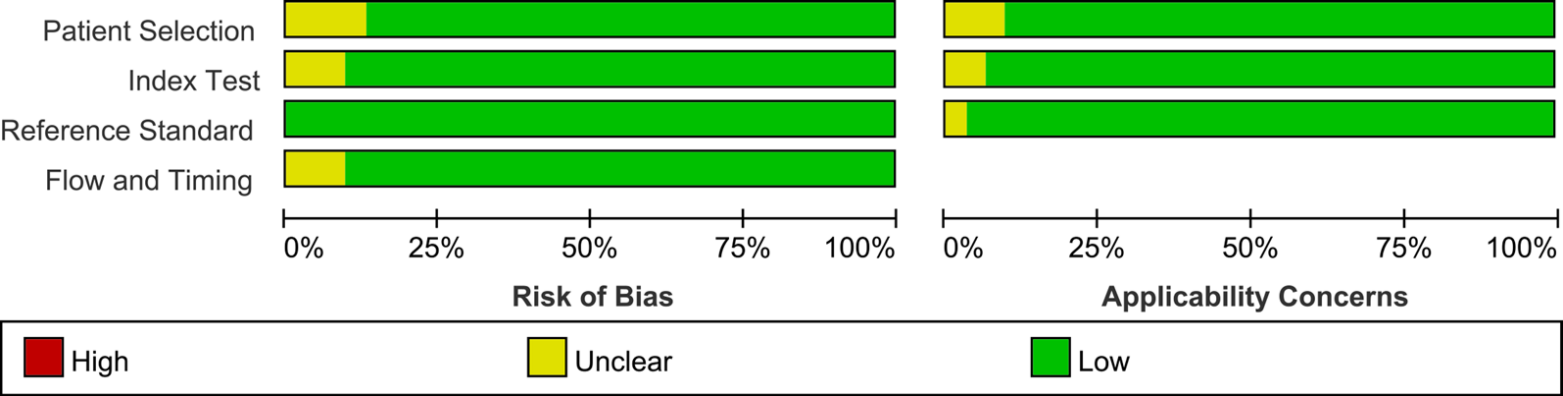
Risks of bias and applicability concerns on the QUADAS-2 tool of the enrolled studies

A total of 367 articles were identified from the databases searched. Among them, 51 duplicates were removed and 254 studies were excluded through an initial screening. After a full text assessment for eligibility of the remaining62 articles, 31 articles with 37 studies and 2585 patients with confirmed or suspected CA were identified for inclusion in this meta-analysis. The articles of Gillmore et al*.* and Kircher et al*.* reported performance evaluation of 3 modalities, respectively. The publications of Lee et al*.* and Minamimoto et al*.* reported results of 2 imaging tools, respectively. No additional studies were found through reference screening of the included papers. Figure 1 shows the flow of the database search and literature selection process. The quality of the included studies was regarded as high according to the QUADAS-2 scale (Fig. 2). Table 1 details the characteristics of studies included.

**Table 1:** Study characteristics

References	Year of publication	No. of participants	Men (%)	Age (SD or IQR)	Population	Study design	Reference test	Modalities	Image analysis	Tracers
Abulizi et al. [44]	2019	27	70	70 (12)	Known or suspected CA	Prospective	Myocardial histology	PET	Semiquantitative	^18^F-NaF
Aquaro et al. [45]	2014	79	61	69 (10)	Known CA	Prospective	Myocardial histology electrocardiographic criteria	CMR	Qualitative	NA
Asif et al. [46]	2020	133	53	76 (12)	Suspected CA	Retrospective	Myocardial histology	SPECT	Semiquantitative	^99m^Tc-PYP
Awaya et al. [47]	2020	10	70	61 (12)	Suspected CA	Prospective	Myocardial histology	SPECT	Qualitative	^99m^Tc-aprotinin
Baggiano et al. [48]	2020	436	66	67 (13)	Suspected CA	Prospective	Myocardial histology	CMR	Qualitative	NA
Baroni et al. [49]	2018	21	74	58 (12)	Suspected CA	Prospective	Myocardial histology	CMR	Qualitative	NA
Bellevre et al. [50]	2020	30	53	84 (7)	Suspected CA	Retrospective	Myocardial histology	SPECT	Semiquantitative	^99m^Tc-HMDP
Bhatti et al. [51]	2016	126	64	63 (10)	Suspected CA	Retrospective	Myocardial histology	CMR	Qualitative	NA
Cappelli et al. [52]	2019	85	79	77 (9)	Suspected CA	Retrospective	Myocardial histology	SPECT	Semiquantitative	^99m^Tc-HMDP
Ezawa et al. [53]	2018	18	61	51 (15)	Known or suspected CA	Prospective	Myocardial histology	PET	Qualitative	^11^C-PIB
Flaherty et al. [54]	2020	43	7	77 (9)	Suspected CA	Prospective	Myocardial histology	SPECT	Quantitative	^99m^Tc-PYP
Gallini et al. [55]	2019	76	80	77 (8)	Known or suspected CA	Retrospective	Myocardial histology	SPECT	Semiquantitative	^99m^Tc-HMDP
Gillmore et al. [56]	2016	374	NR	NR	Known or suspected CA	Prospective	Myocardial histology and genetic findings	SPECT	Qualitative	^99m^Tc-DPD
Karamitsos et al. [57]	2013	53	66	62 (11)	Known or suspected CA	Prospective	Myocardial histology	CMR	Qualitative	NA
Kircher et al. [58]	2019	21	64	65 (14)	Suspected CA	Prospective	Myocardial histology	CMR	Qualitative	NA
Lee et al. [59]	2015	19	46	65 (10)	Suspected CA	Prospective	Myocardial histology	CMR	Qualitative	NA
Malka et al. [38]	2020	308	75	73 (8)	Known CA and controls	Retrospective	Myocardial histology	SPECT	Semiquantitative	^99m^Tc-HMDP
Martineau et al. [60]	2019	15	80	69 (11)	CA	Retrospective	Myocardial histology	PET	Qualitative	^18^F-NaF
Masri et al. [61]	2020	233	69	77 (14)	Suspected CA	Prospective	Diffuse myocardial uptake	SPECT	Semiquantitative	^99m^Tc-PYP
Minamimoto et al. [16]	2020	9	67	64 (14)	Suspected CA	Prospective	Myocardial histology	SPECT	Qualitative	^99m^Tc-aprotinin
Moore et al. [62]	2017	21	91	NR	Suspected CA	Prospective	Myocardial histology	SPECT	Semiquantitative	^99m^Tc-DPD
Papantoniou et al. [63]	2015	12	67	69 (12)	Suspected CA	Prospective	Myocardial histology	SPECT	Semiquantitative	^99m^Tc-PYP
Papathanasiou et al. [42]	2020	17	88	71 (9)	Known CA and controls	Retrospective	Myocardial histology	PET	Qualitative	^18^F-flutemetamol
Poterucha et al. [64]	2020	91	84	72 (9)	Suspected CA	Retrospective	Myocardial histology	SPECT	Qualitative	^99m^Tc-PYP
Rapezzi et al. [65]	2011	63	62	53 (41–66)	Known or suspected CA	Prospective	Myocardial histology	SPECT	Qualitative	^99m^Tc-DPD
Régis et al. [66]	2020	40	73	75 (10)	Suspected CA	Retrospective	H/CL ratio	SPECT	Qualitative	^99m^Tc-PYP
Rosengren et al. [41]	2020	51	73	69 (13)	Known and suspected CA	Prospective	Myocardial histology	PET	Qualitative	^11^C-PIB
Sperry et al. [37]	2020	100	75	77 (72–82)	Suspected CA	Retrospective	Semiquantitative grade and H/CL ratio	SPECT	Semiquantitative	^99m^Tc-PYP
White et al. [67]	2014	25	58	62 (13)	Suspected CA	Prospective	Myocardial histology	CMR	Semiquantitative	NA
Wollenweber et al. [39]	2020	32	722	73 (11)	Suspected CA	Prospective	Myocardial histology	SPECT	Qualitative	^99m^Tc-DPD
Zhang et al. [40]	2020	17	94	77 (8)	Known and suspected CA	Prospective	Myocardial histology	PET	Qualitative	^18^F-NaF

*CA* cardiac amyloid, *SD* standard deviation, *IQR* interquartile range, *CMR* cardiac magnetic resonance, *SPECT* single photon emission computed tomography, *PET* positron emission tomography, *NR* not reported, *NA* not applicable

### Diagnostic performance of noninvasive modalities

**Fig. 3:**
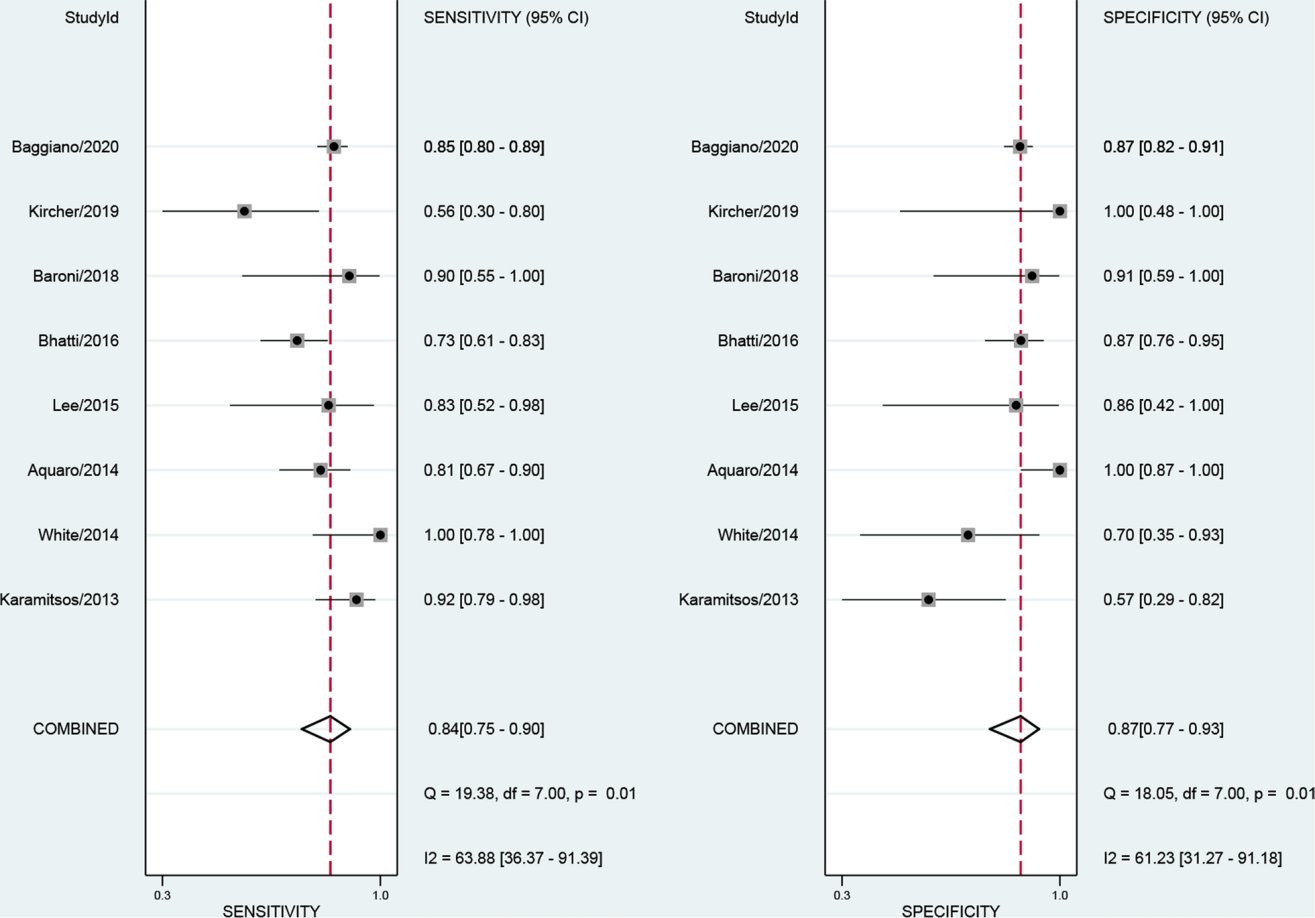
Forest plot for diagnostic performance of CMR

**Fig. 4:**
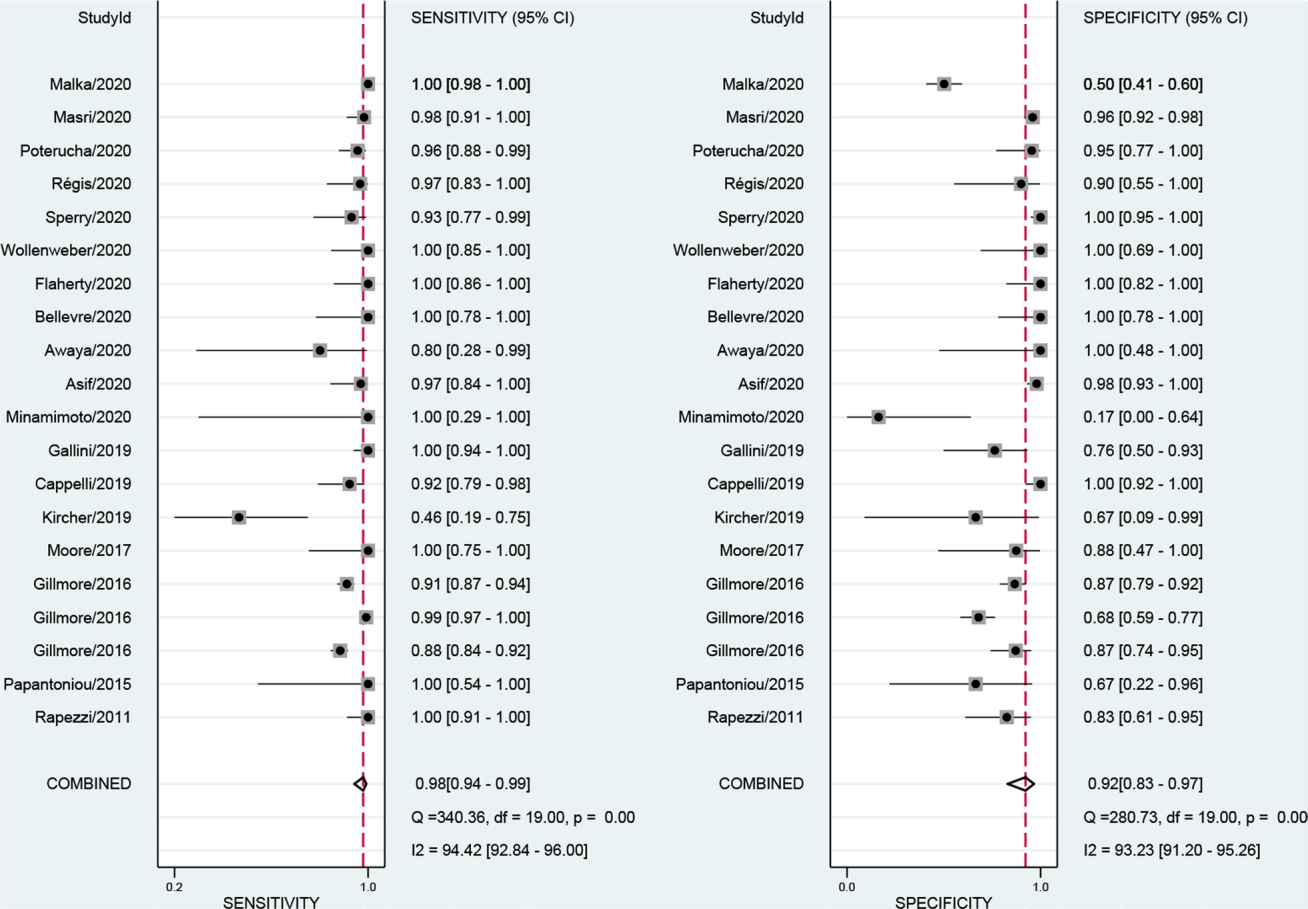
Forest plot for diagnostic performance of SPECT

**Fig. 5:**
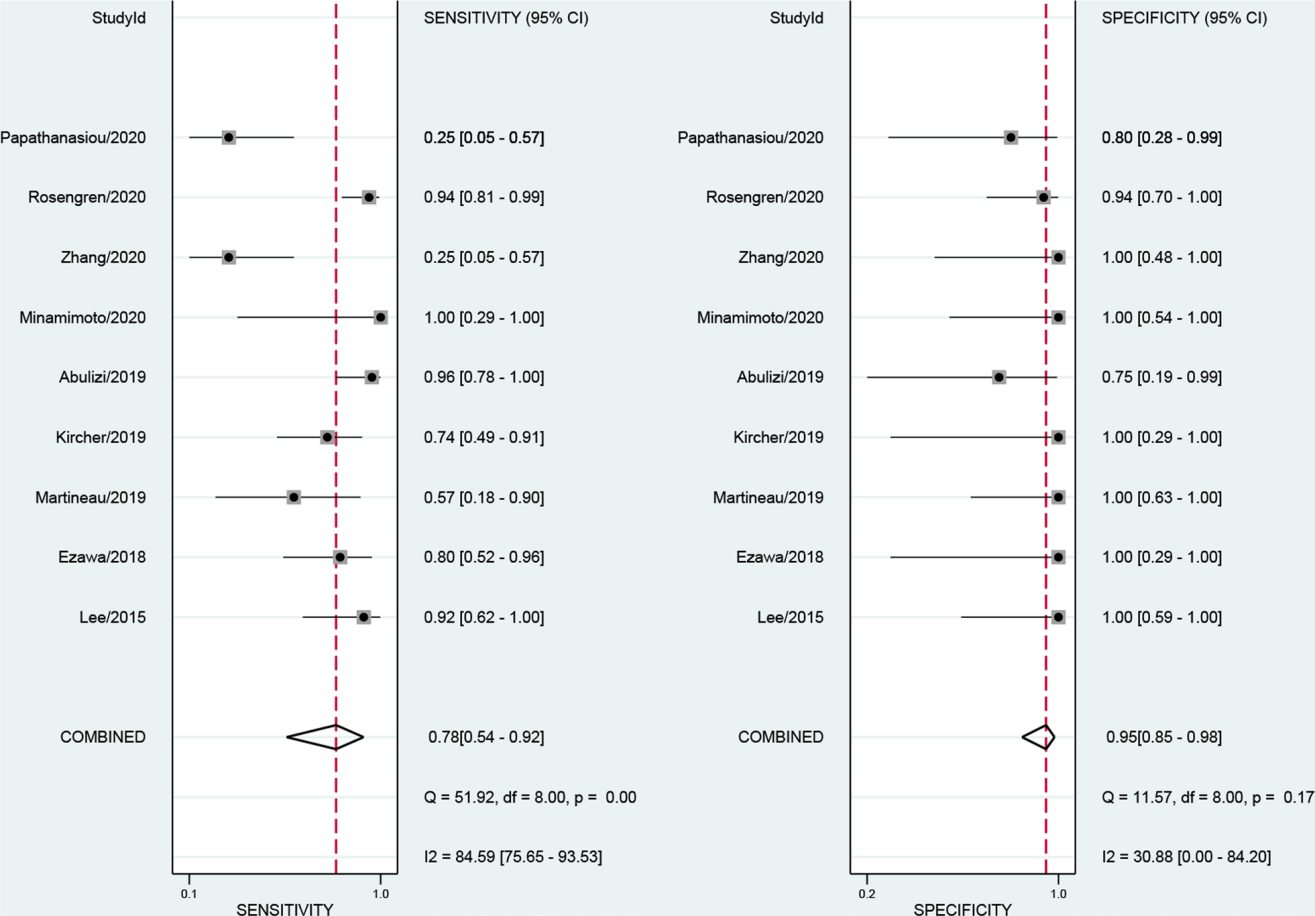
Forest plot for diagnostic performance of PET

The numbers of studies included in the analysis of CMR, SPECT and PET were 8, 20 and 9, respectively. The pooled sensitivity of CMR, SPECT and PET were 0.84 [0.75, 0.90], 0.98 [0.94, 0.99] and 0.78 [0.54, 0.92], respectively. The overall specificities were 0.87 [0.77, 0.93], 0.92 [0.83, 0.97] and 0.95 [0.85, 0.98] for CMR, SPECT and PET, respectively (Figs. 3, 4, 5). The AUC values of CMR, SPECT and PET were 0.92 [0.89, 0.94], 0.99 [0.98, 1.00] and 0.95 [0.93, 0.96].

### Diagnostic performance of prospective studies

With regard to prospective studies of these detection approaches, the respective overall sensitivities of CMR, SPECT and PET were 0.85 [0.76, 0.91], 0.98 [0.90, 0.99] and 0.85 [0.63, 0.95]. The pooled specificities were 0.89 [0.72, 0.96], 0.87 [0.73, 0.94] and 0.98 [0.68, 1.00] for CMR, SPECT and PET, respectively (Additional file 1: Figure S1, Additional file 2: Figure S2, Additional file 3: Figure S3). The AUC values of CMR, SPECT and PET were 0.92 [0.89, 0.94], 0.97 [0.96, 0.98] and 0.98 [0.97, 0.99].

### Subgroup analysis of SPECT tracers

The numbers of studies using ^99m^Tc-DPD, ^99m^Tc-PYP, ^99m^Tc-HMDP, and ^99m^Tc-aprotinin for SPECT radiotracers were 5, 8, 5, and 2, respectively. Studies using ^99m^Tc-aprotinin were not enrolled in pooled analysis for the inadequate number of studies. Overall results demonstrated that ^99m^Tc-HMDP manifested the highest sensitivity (0.99 [0.83, 1.00]). ^99m^Tc-PYP had the highest pooled specificity (0.95 [0.86, 0.99]). The pooled sensitivity of ^99m^Tc-DPD and ^99m^Tc-PYP reached 0.98 (Additional file 4: Figure S4, Additional file 5: Figure S5, Additional file 6: Figure S6). The AUC values of ^99m^Tc-DPD, ^99m^Tc-PYP, ^99m^Tc-HMDP were 0.89 [0.86, 0.92], 0.99 [0.98, 1.00], and 0.99[0.98, 1.00], respectively.

### Subgroup analysis of PET tracers

The number of included studies using ^11^C-PIB, ^18^F-florbetaben, ^18^F-flutemetamol, and ^18^F-NaF for PET tracers were 4, 1, 1, and 3, respectively. Only PET studies utilizing ^11^C-PIB were included in pooled analysis. It demonstrated a pooled sensitivity of 0.91 [0.81, 0.96], and its pooled specificity was 0.97 [0.81, 1.00] (Additional file 7: Figure S7). The AUC value of ^11^C-PIB was 0.98 [0.97, 0.99]. Both the reported sensitivity and specificity of ^18^F-florbetaben PET for the separation of patients with CA from patients without CA were 100%.The study of ^18^F-flutemetamol showed a sensitivity of 0.17 with a high proportion of false-negative PET results.

### Heterogeneity and publication bias

**Fig. 6:**
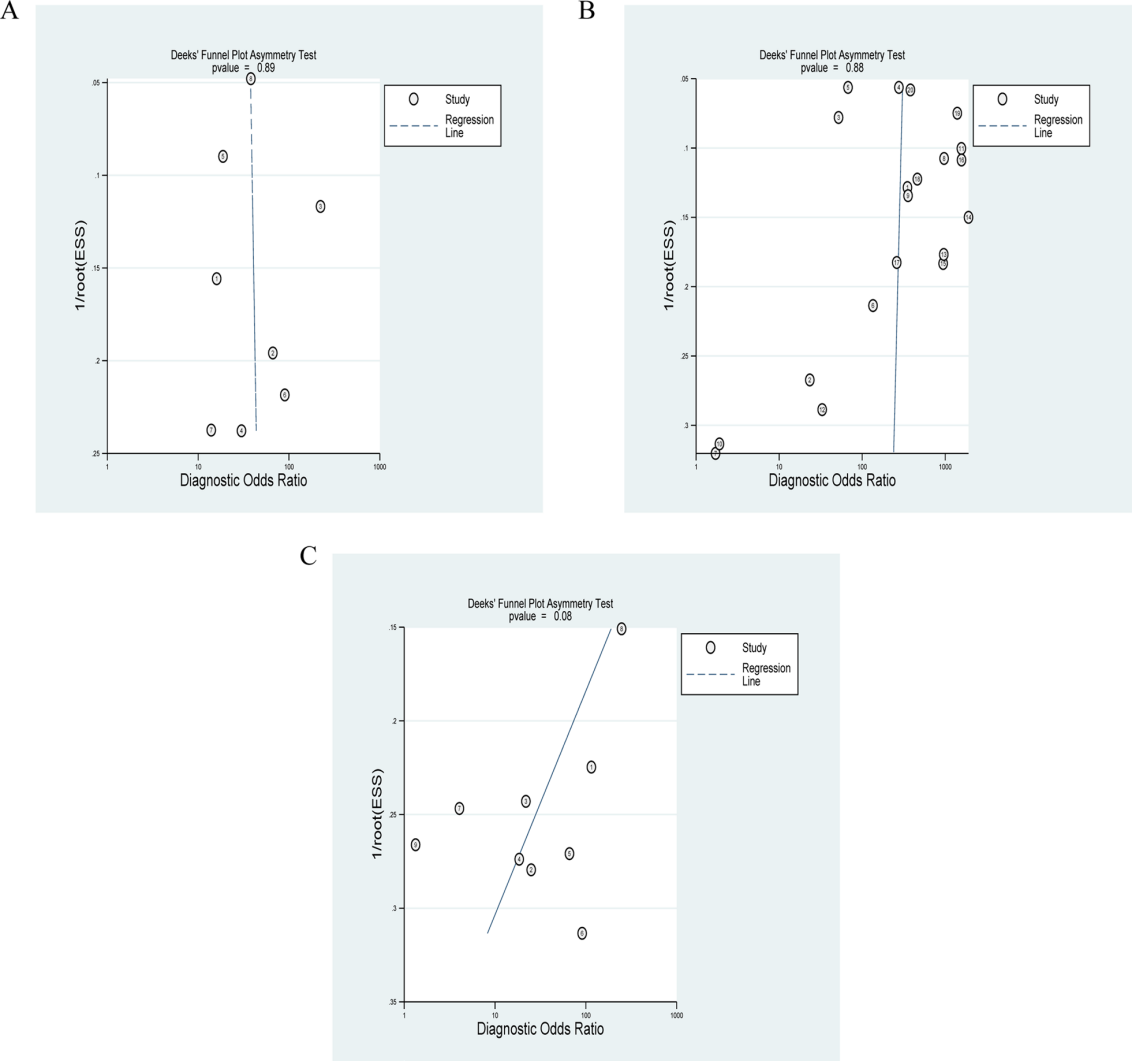
Funnel plots for diagnostic performance of CMR, SPECT and PET. **A** Funnel plot for diagnostic performance of CMR. **B** Funnel plot for diagnostic performance of SPECT. **C** Funnel plot for diagnostic performance of PET

The I^2^ values for meta-analysis of CMR were 64 (pooled sensitivity) and 61 (pooled specificity). The respective I^2^ static for SPECT were 94 and 93. As for PET, the I^2^ values for pooled analysis of sensitivity and pooled specificity were 85 and 31. Deek’s funnel plot asymmetry tests for publication bias yielded p values of 0.89, 0.88, and 0.08 for CMR, SPECT and PET, which revealed that there may be no potential publication bias in the study (Fig. 6).

### Sensitivity analysis

Sensitivity analysis was conducted to assess the potential influence of single study on the overall results. After omitting each study one by one, the pooled results of CMR, SPECT, PET and the corresponding subgroup analysis remained robust (Additional file 8: Figure S8, Additional file 9: Figure S9, Additional file 10: Figure S10).

## Discussion

CA is part of systemic amyloidosis, it’s characterized by the abnormal accumulation of amyloid fibrils within the extracellular of the myocardial tissue [28]. Accurate and timely confirmation of CA is of particular importance because cardiac involvement usually can be lethal [29]. Endomyocardial biopsy remains the gold standard for the detection and evaluation of prognosis of CA [30]. However, it’s an invasive method and introduces potential damage to human body [31, 32]. Among those noninvasive modalities, cardiac ultrasound is widely used, but the diagnostic accuracy is relatively low, and it is clinically used to identify potential patients with CA and further workup should be conducted [33, 34]. It is reported that CMR manifested favorable sensitivity and specificity in the identification of CA regardless of its low cost-effectiveness [10, 35]. The increase in myocardial extracellular volume (ECV) is readily detected by CMR via the Late Gadolinium Enhancement (LGE) test, which demonstrated a sensitivity of 80% and a specificity of 90% in detecting CA [34, 36]. Furthermore, the administration of SPECT scans with ^99m^Tc-DPD, ^99m^Tc-PYP, ^99m^Tc-HMDP revealed promising results [37–39]. Compared with SPECT, PET showed higher spatial resolution, it has been represented as a promising approach in the field of CA diagnosis [40–42]. In clinical setting, each single or the combination employment of the above cardiac imaging approaches need to be explained together with the other clinical findings. The imaging techniques not only help to diagnose CA, but also help to estimate the type and the severity of the disease, provide prognostic markers of the disease and monitor the effectiveness of therapy [43]. This meta-analysis is focused on the role of the first of these steps: diagnosis of CA.

Previous meta-analysis commonly focused on single diagnosis tool of CA [19, 21–23]. We conducted a meta-analysis to directly compare the performance of CMR, SPECT and PET for the diagnosis of CA. The analysis was on the updated articles with respect to study design, type of radiotracers in SPECT and PET scans. This is one of the strengths of this study. It is worth noting 20 of the total 31 articles included in this meta-analysis were published in the years of 2019 and 2020, which indicated that noninvasive diagnostic modalities especially SPECT and PET scans have been extensively investigated. In general, results of this meta-analysis revealed that CMR, SPECT, and PET presented high sensitivity and specificity for the diagnosis of CA. The pooled sensitivity (0.98 [0.94, 0.99]) of SPECT scan was the highest. PET manifested the highest pooled specificity (0.95 [0.85, 0.98]). The AUC values of CMR, SPECT and PET were 0.92 [0.89, 0.94], 0.99 [0.98, 1.00] and 0.95 [0.93, 0.96], respectively. When prospective studies were considered, overall sensitivity of SPECT was still the highest (0.98 [0.90, 0.99]). Interestingly, PET scans showed the highest specificity (0.98 [0.68, 1.00]). On the basis of this difference in results, we can make a preliminary conclusion that the study design could be the source of heterogeneity of enrolled studies. Besides, results manifested ^99m^Tc-HMDP had the highest sensitivity (0.99 [0.83, 1.00]), ^99m^Tc-PYPhad the highest pooled specificity (0.95 [0.86, 0.99]). ^99m^Tc-PYP and ^99m^Tc-HMDP revealed good diagnostic performance with AUC values of 0.99 [0.98, 1.00] and 0.99 [0.98, 1.00], respectively. As for PET scans, PET studies using ^11^C-PIB was included in pooled analysis, both the pooled sensitivity and specificity reached more than 0.90, the AUC value of was surprisingly 0.98. One study reported that the sensitivity and specificity of ^18^F-florbetaben PET for the detection of CA were 100%, the level of evidence in this study was relatively lower than a meta-analysis, and therefore a possibly pooled analysis of PET scans using ^18^F-florbetaben is recommended in the future.

In this meta-analysis, we comprehensively searched the online database to enhance the possibility of retrieving as more eligible studies as we could. Two researchers independently performed the whole process of information extraction under the guidance of the study protocol. Moreover, the heterogeneity across the studies included was assessed using Cochran Q test. In general, there existed significant heterogeneities among studies. The sources of heterogeneity may be attributed to difference in the year of publication, study design (as mentioned above), and patient characteristics. We indented to conduct meta-regression to explore the possible origins of heterogeneity, unfortunately, the numbers of PET and CMR studies were insufficient to complete meta-regression. The underlying sources of heterogeneity would be investigated in further studies. Moreover, results of sensitivity analysis claimed that after omitting individual study one after another, the pooled indicators were robust in this study. The Deek’s funnel plot asymmetry tests for publication bias revealed that there may not be publication bias in the meta-analysis. Despite the existence of heterogeneity, we may conclude based on the pooled results that this analysis could provide evidence-based information for scientific research and practical applications in the process of CA diagnosis. As far as scientific research is concerned, prospective studies and PET radiotracers with higher spatial resolution need to be further investigated on the basis of results of this meta-analysis. Meta-analysis with larger sample-sized and amount of studies are recommended. With regard to applications in clinical settings, decision-making of practitioners in the diagnosis of CA should be made according to technical merit, consideration of cost-effectiveness, and the availability of specific modalities. In order to enhance diagnostic accuracy of CA, if possible, the combination of different diagnostic tools is recommended.

## Supplementary Information

**Additional file 1.** Forest plot for diagnostic performance of CMR in prospective studies Forest plot for diagnostic performance of CMR in prospective studies.

**Additional file 2.** Forest plot for diagnostic performance of SPECT in prospective studies Forest plot for diagnostic performance of SPECT in prospective studies.

**Additional file 3.** Forest plot for diagnostic performance of PET in prospective studies Forest plot for diagnostic performance of PET in prospective studies.

**Additional file 4.** Forest plot for diagnostic performance of 99mTc-DPD SPECT Forest plot for diagnostic performance of ^99m^Tc-DPD SPECT.

**Additional file 5.** Forest plot for diagnostic performance of 99mTc-PYP SPECT Forest plot for diagnostic performance of ^99m^Tc-PYP SPECT.

**Additional file 6.** Forest plot for diagnostic performance of 99mTc-HMDP SPECT Forest plot for diagnostic performance of ^99m^Tc-HMDP SPECT.

**Additional file 7.** Forest plot for diagnostic performance of 11C-PIB PET Forest plot for diagnostic performance of ^11^C-PIB PET.

**Additional file 8.** Results of sensitivity analysis of CMR imaging Results of sensitivity analysis of CMR imaging.

**Additional file 9.** Results of sensitivity analysis of SPECT imaging Results of sensitivity analysis of SPECT imaging.

**Additional file 10.** Results of sensitivity analysis of PET imaging Results of sensitivity analysis of PET imaging.

## Data Availability

The datasets used and/or analysed during the current study are available from the corresponding author on reasonable request.

## Abbreviations

CA: Cardiac amyloidosis
AL: Systemic light chain amyloidosis
ATTR: Transthyretin amyloidosis
CMR: Cardiac magnetic resonance
SPECT: Single photon emission computed tomography
PET: Positron emission tomography
CT: Computerized tomography
PRISMA: Preferred Reporting Items for Systematic Reviews and Meta-analysis
TP: True positive
FP: False positive
TN: True negative
FN: False negative
PPV: Positive predictive value
NPV: Negative predictive value
DOR: Diagnostic odds ratio
CI: Confidence interval
SROC: Summary receiver operating characteristic
AUC: Area under the SROC curve
QUADAS-2: Quality Assessment of Diagnostic Accuracy Studies-2
LGE: Late Gadolinium Enhancement
