# Supplementary figures and images for: Diagnostic performance of CMR, SPECT, and PET imaging for the detection of cardiac amyloidosis: a meta-analysis
Source: BMC Cardiovasc Disord. 2021 Oct 7;21:482. doi: 10.1186/s12872-021-02292-z (PMC8499558; doi:10.1186/s12872-021-02292-z)

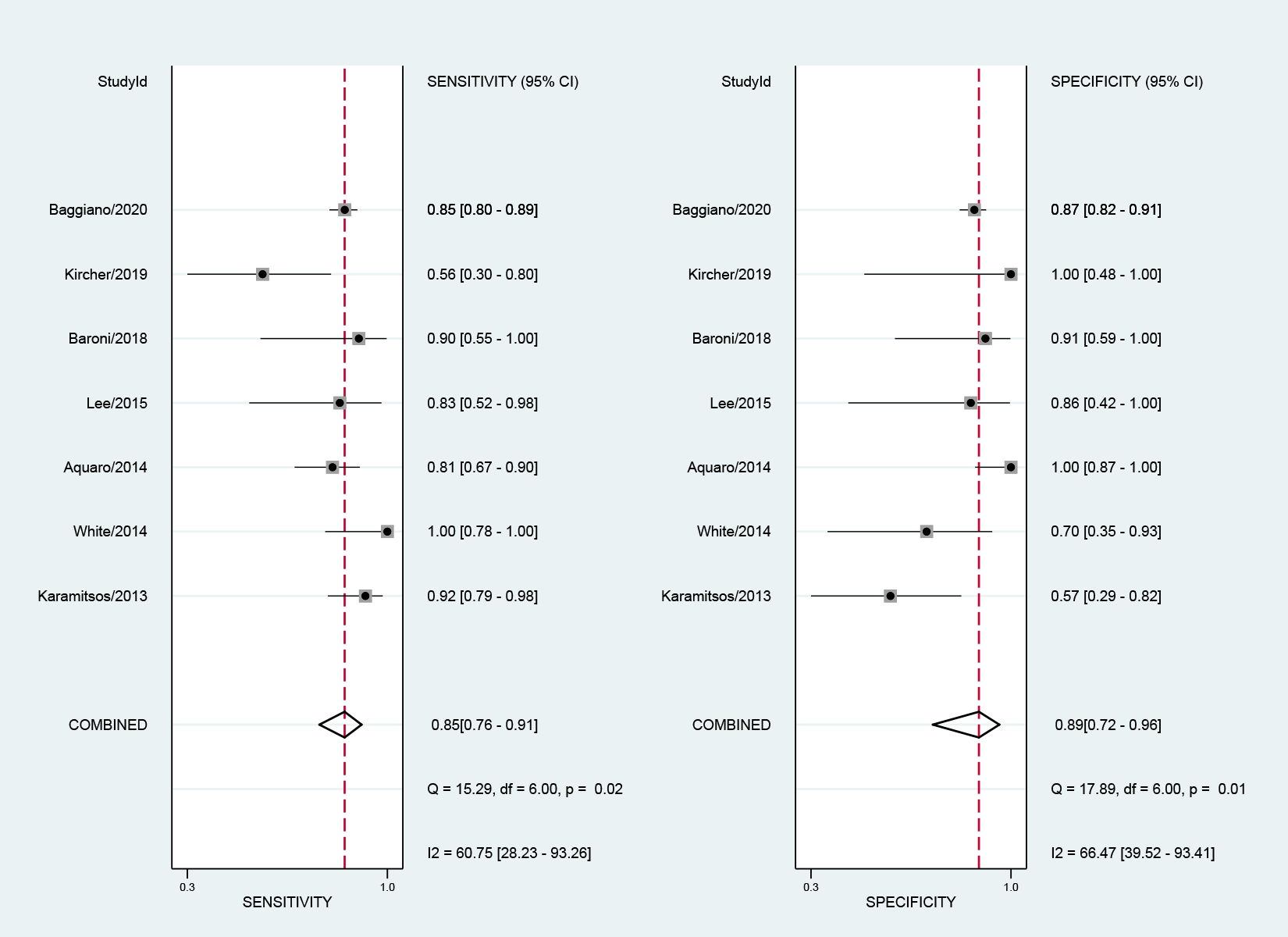

Supplement: Supplementary file 1 — Additional file 1. Forest plot for diagnostic performance of CMR in prospective studies Forest plot for diagnostic performance of CMR in prospective studies. [file 12872_2021_2292_MOESM1_ESM.tif]

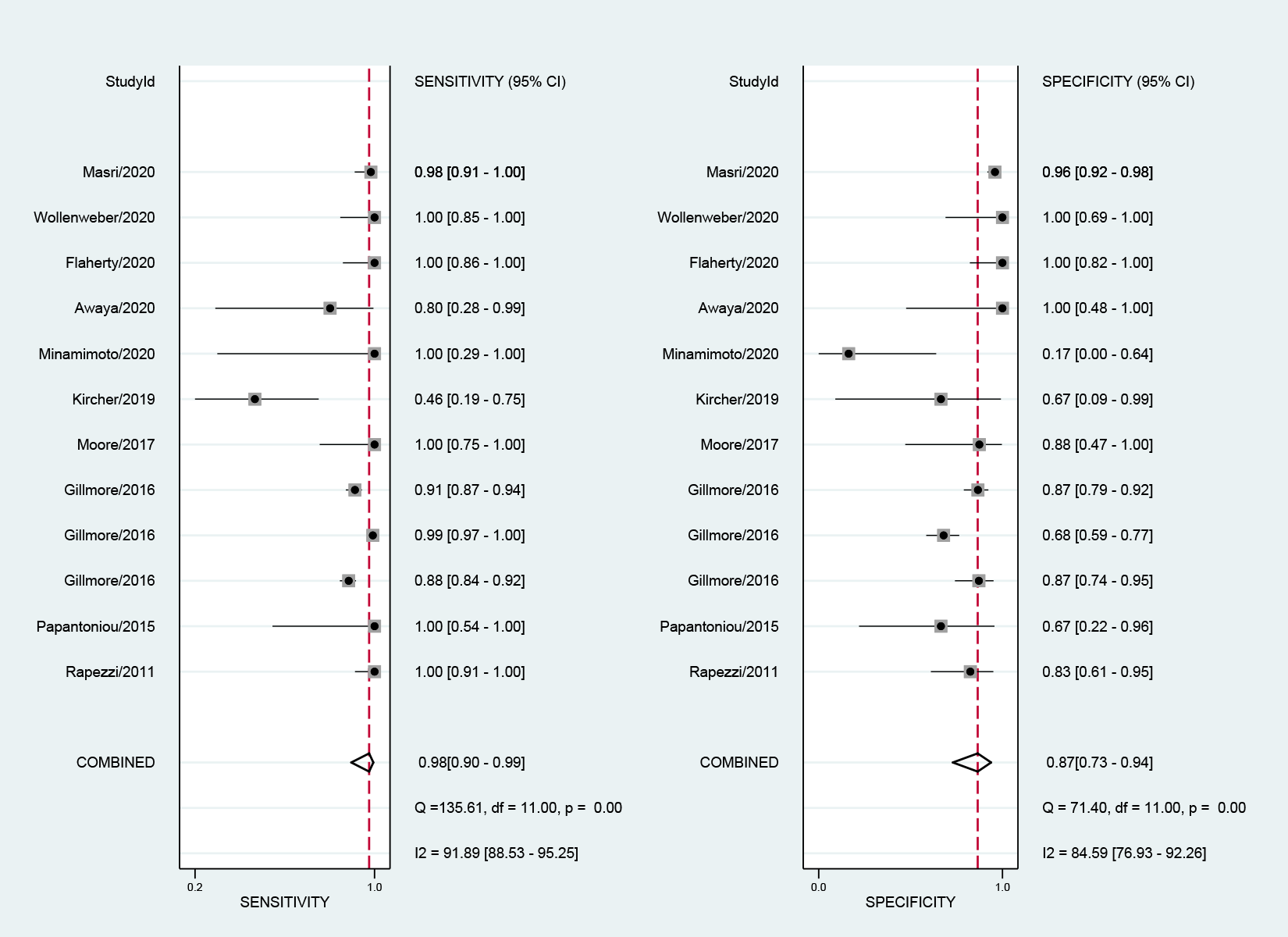

Supplement: Supplementary file 2 — Additional file 2. Forest plot for diagnostic performance of SPECT in prospective studies Forest plot for diagnostic performance of SPECT in prospective studies. [file 12872_2021_2292_MOESM2_ESM.tif]

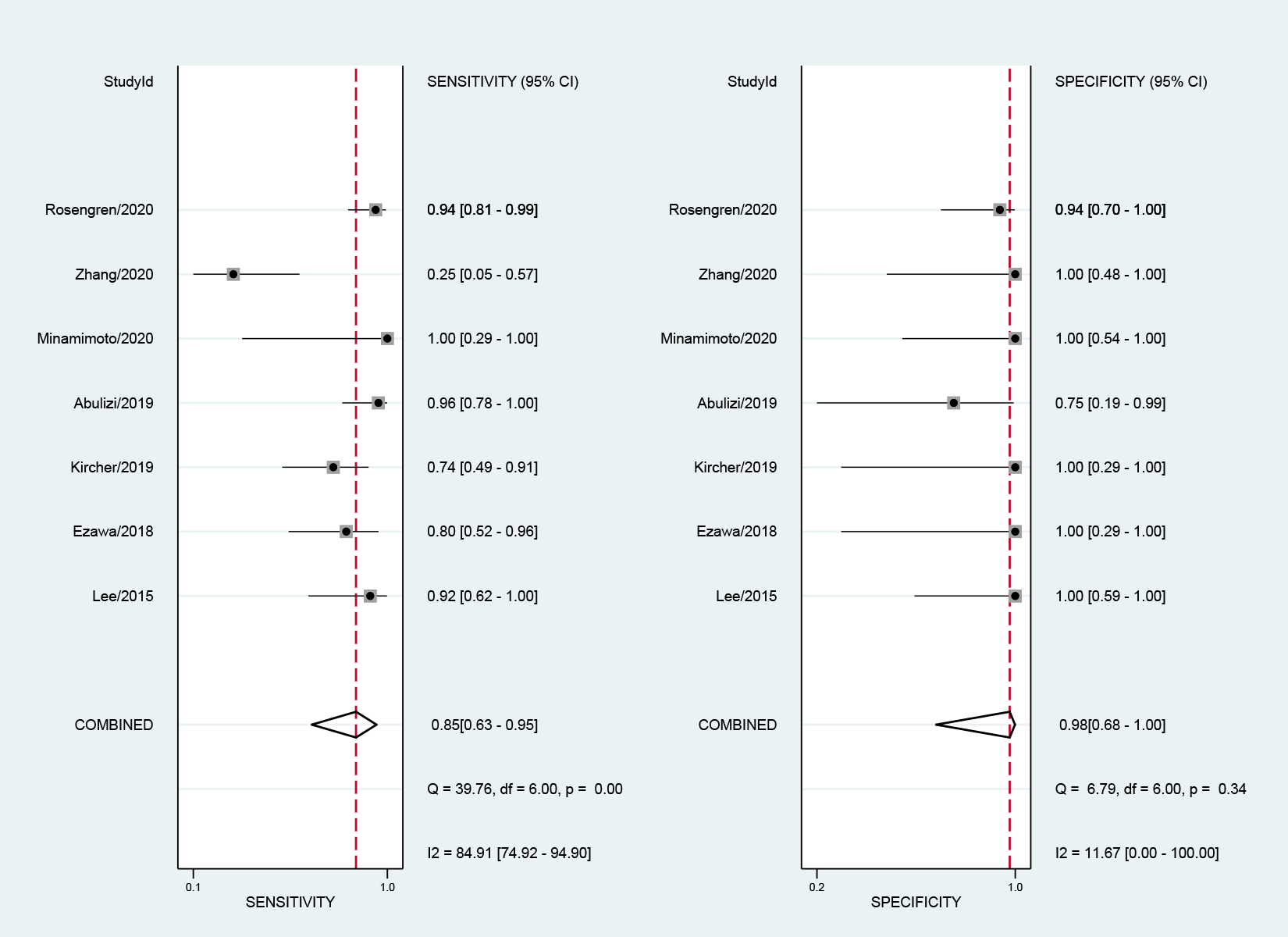

Supplement: Supplementary file 3 — Additional file 3. Forest plot for diagnostic performance of PET in prospective studies Forest plot for diagnostic performance of PET in prospective studies. [file 12872_2021_2292_MOESM3_ESM.tif]

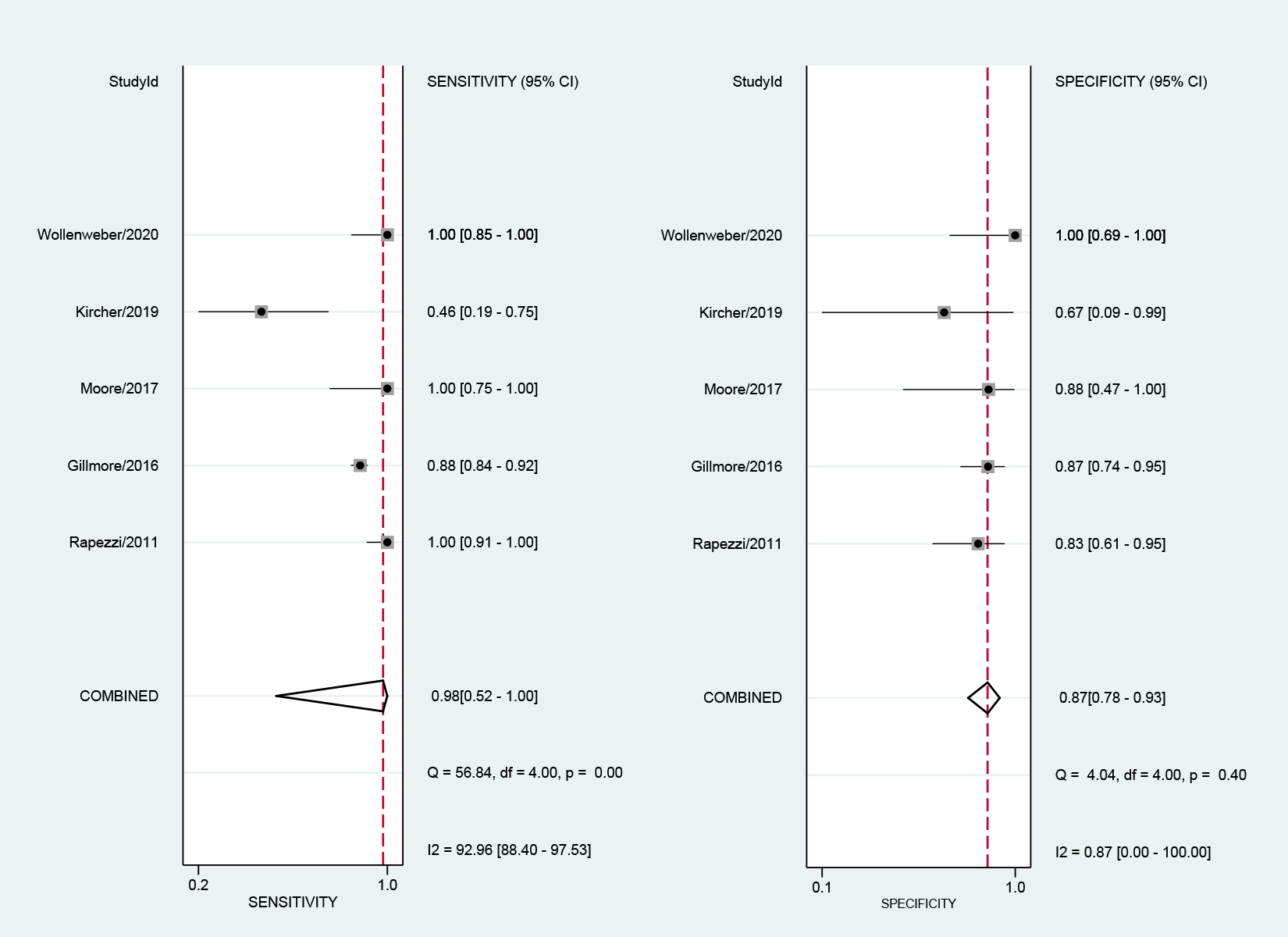

Supplement: Supplementary file 4 — Additional file 4. Forest plot for diagnostic performance of 99mTc-DPD SPECT Forest plot for diagnostic performance of 99mTc-DPD SPECT. [file 12872_2021_2292_MOESM4_ESM.tif]

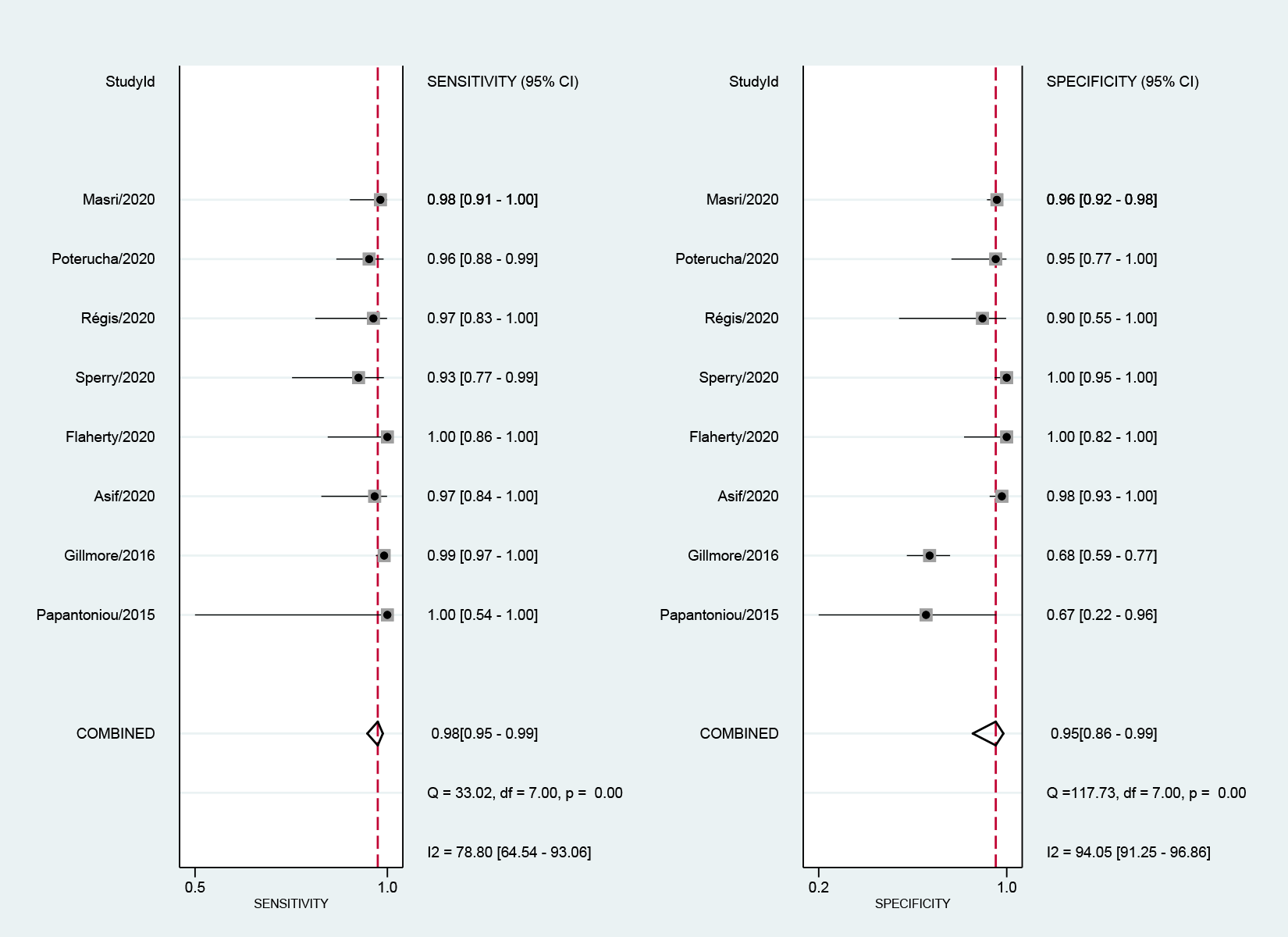

Supplement: Supplementary file 5 — Additional file 5. Forest plot for diagnostic performance of 99mTc-PYP SPECT Forest plot for diagnostic performance of 99mTc-PYP SPECT. [file 12872_2021_2292_MOESM5_ESM.tif]

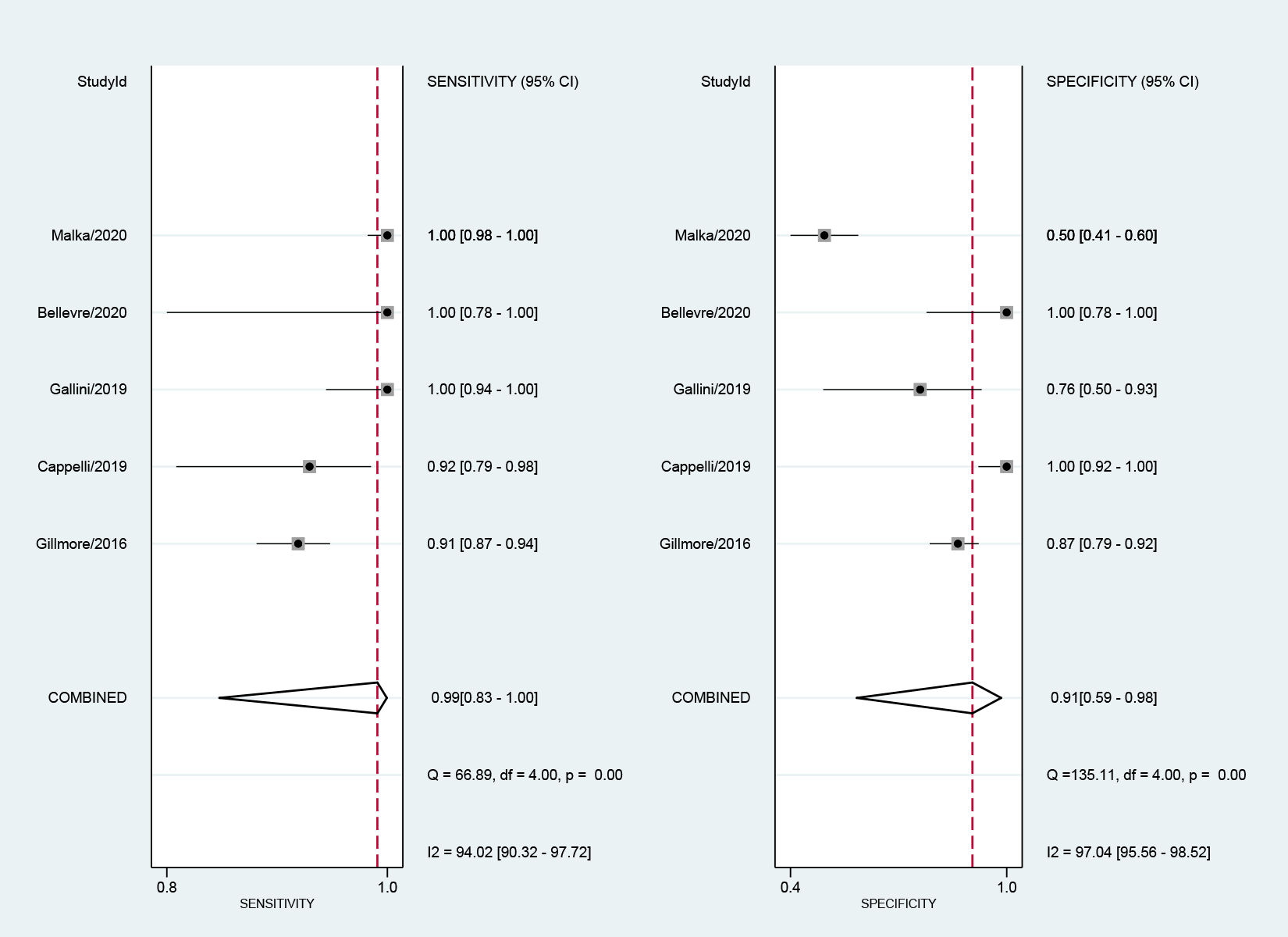

Supplement: Supplementary file 6 — Additional file 6. Forest plot for diagnostic performance of 99mTc-HMDP SPECT Forest plot for diagnostic performance of 99mTc-HMDP SPECT. [file 12872_2021_2292_MOESM6_ESM.tif]

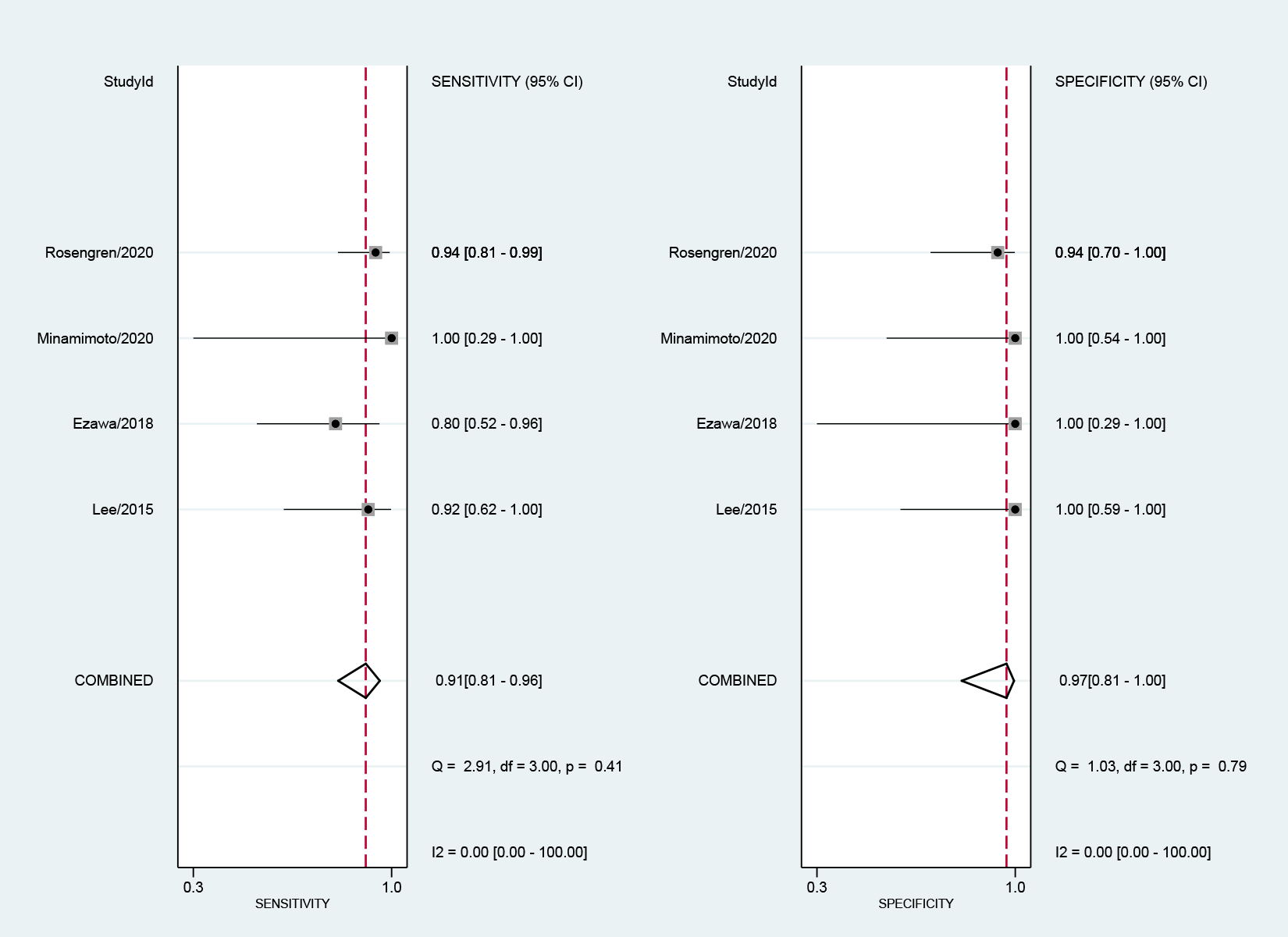

Supplement: Supplementary file 7 — Additional file 7. Forest plot for diagnostic performance of 11C-PIB PET Forest plot for diagnostic performance of 11C-PIB PET. [file 12872_2021_2292_MOESM7_ESM.tif]

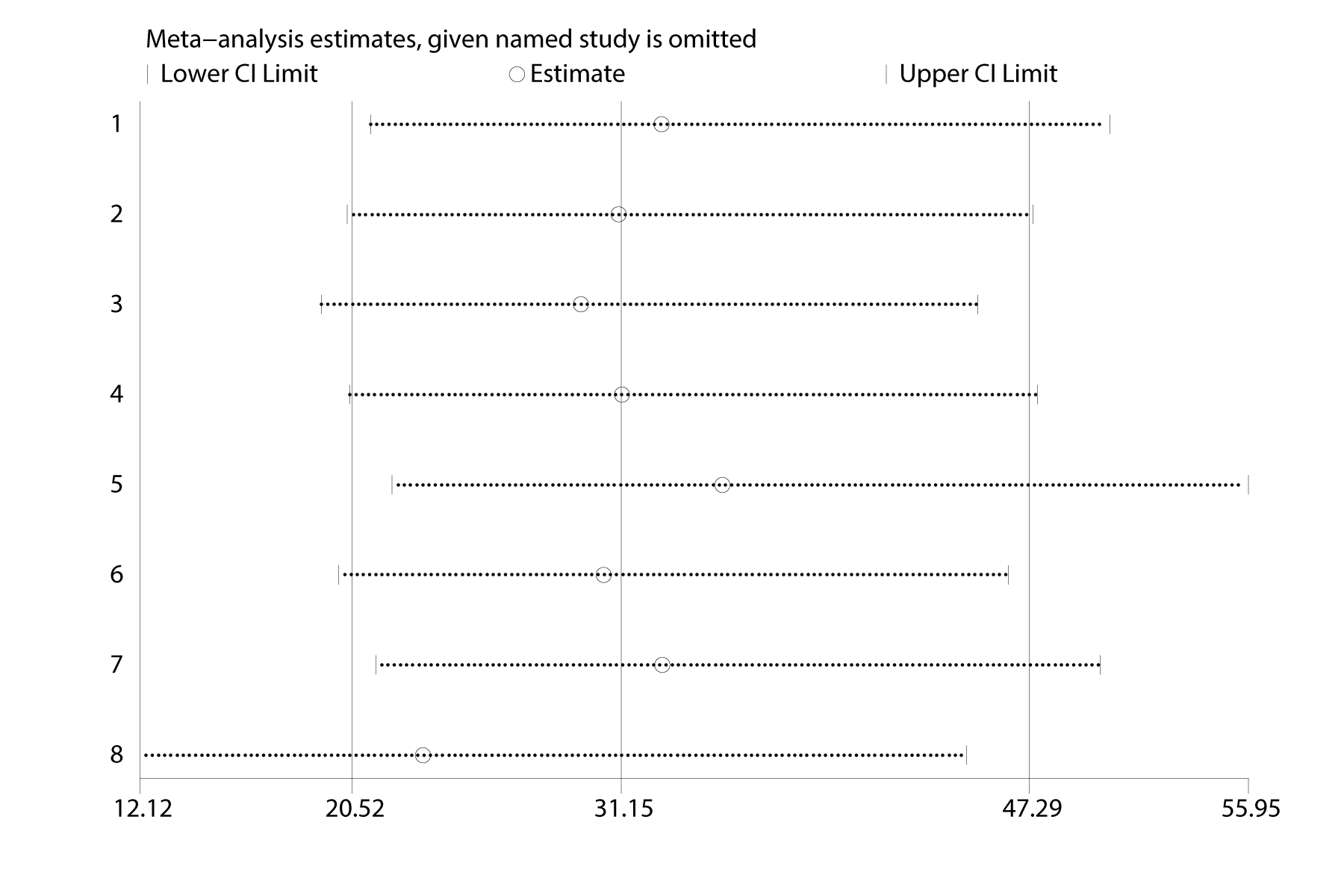

Supplement: Supplementary file 8 — Additional file 8. Results of sensitivity analysis of CMR imaging Results of sensitivity analysis of CMR imaging. [file 12872_2021_2292_MOESM8_ESM.tif]

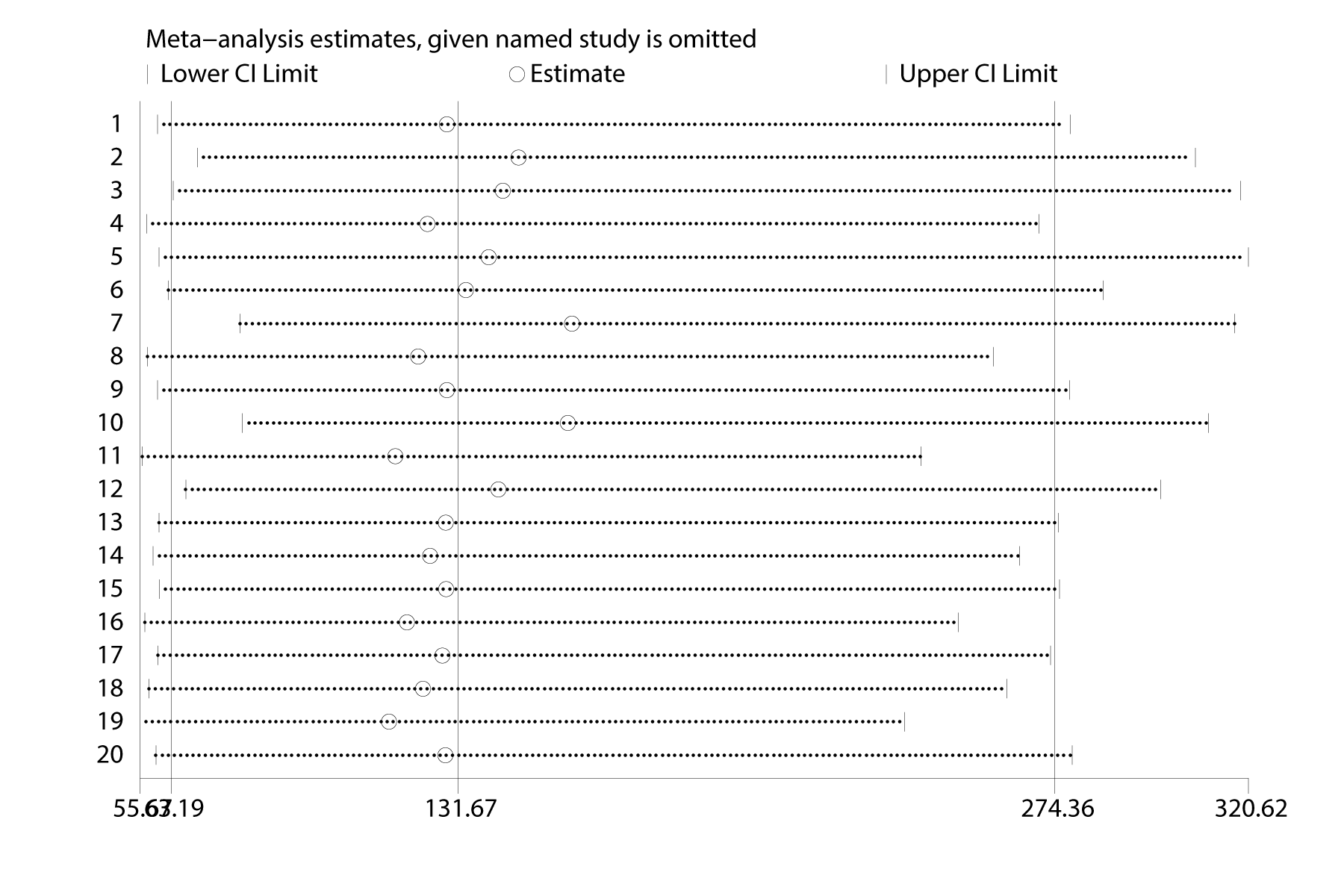

Supplement: Supplementary file 9 — Additional file 9. Results of sensitivity analysis of SPECT imaging Results of sensitivity analysis of SPECT imaging. [file 12872_2021_2292_MOESM9_ESM.tif]

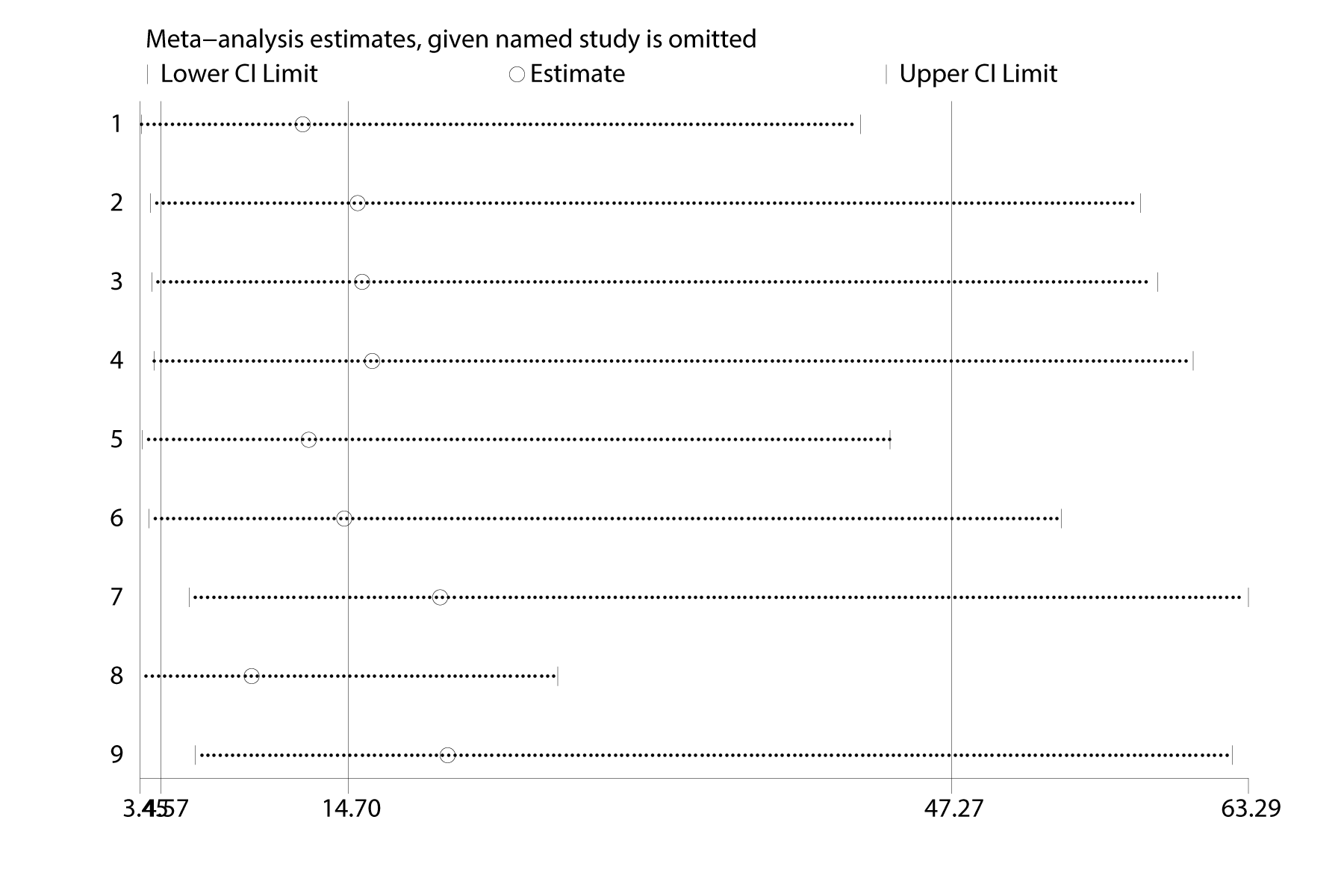

Supplement: Supplementary file 10 — Additional file 10. Results of sensitivity analysis of PET imaging Results of sensitivity analysis of PET imaging. [file 12872_2021_2292_MOESM10_ESM.tif]
